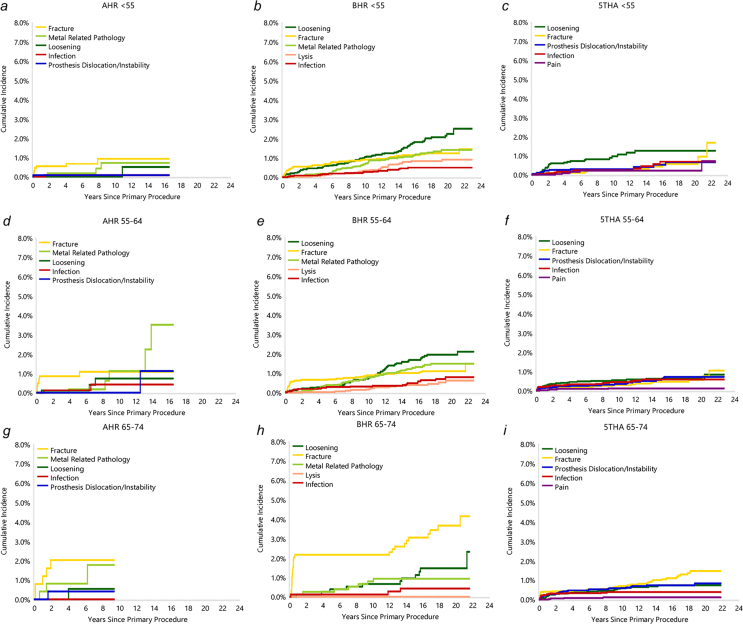# Seventeen-year outcomes for a contemporary total hip resurfacing prosthesis in Australia: an analysis of registry data with comparison to best performing conventional and most prevalent resurfacing prostheses

**DOI:** 10.1016/j.jor.2025.07.012

**Published:** 2025-07-14

**Authors:** Cameron J. Wilson, Michelle F. Lorimer, Carl Holder, James D. Stoney, Patrick C.L. Weinrauch

**Affiliations:** a Independent Writer and Researcher, Buderim, Queensland, Australia; b South Australian Health and Medical Research Institute (SAHMRI), Adelaide, South Australia, Australia; c Australian Orthopaedic Association National Joint Replacement Registry (AOANJRR), Adelaide, South Australia, Australia; d Department of Orthopaedic Surgery, St. Vincent's Hospital, Melbourne, Victoria, Australia; e Brisbane Hip Clinic, Fortitude Valley, Queensland, Australia; f School of Medicine, Gold Coast Campus, Griffith University, Queensland, Australia

**Keywords:** Hip resurfacing arthroplasty, Revision, Primary total hip arthroplasty, Age, Metal-on-metal, Registry

## Abstract

**Background:**

This study addresses survivorship up to 17 years for the hip resurfacing arthroplasty (HRA) system most commonly used in Australia at present. Here we compare overall and age-stratified revision rates of the study HRA to a benchmark HRA and the five conventional prostheses with the lowest 10-year cumulative percent revision (5THA) in the Australian Orthopaedic Association National Joint Replacement Registry (AOANJRR). We further compare outcomes for the resurfacing target cohort of men under 65 years. Finally, we compare revision diagnoses for the study prosthesis and benchmarks.

**Methods:**

AOANJRR data were analysed for osteoarthritis patients who underwent total hip arthroplasty, to the end of 2023. Using Cox proportional hazards models, we compared revision rates (estimated using Kaplan–Meier survival curves with 95 % confidence intervals) across the three groups. Femoral head sizes <50 mm for HRA and <32 mm for 5THA were excluded. Cumulative incidence plots were used to compare revision diagnoses.

**Results:**

For the study HRA, the 17-year survivorship was 94 %, with revision risks comparable to the benchmark HRA but inferior to 5THA. For patients under 55 years, revision rates were comparable to those of 5THA and superior to the benchmark HRA. Revision risks were not significantly different between the three groups for men under 65 (95 % survivorship for the study HRA). Fracture and metal-related pathology were the most likely reasons for early and late revision respectively, while loosening and fracture were the most common revision diagnoses for the benchmark HRA and 5THA respectively.

**Conclusion:**

The study HRA achieved similar survivorship to the benchmark HRA and 5THA in men <65 years, but inferior to 5THA overall. Outcomes were best for younger patients. Adverse metal reactions remain a concern, with longer follow-up essential to assess their impacts and other trends in revision diagnoses.

## 1 Introduction

Total hip resurfacing arthroplasty (HRA) using metal-on-metal bearings has proposed benefits for younger, more active patients likely to outlive their hip prosthesis.^1^, ^2^, ^3^, ^4^ It was intended to defer the need for total hip arthroplasty (THA) and its conservation of bone stock theoretically facilitates this subsequent conversion,^5^ although this has not necessarily translated into improved revision outcomes.^6^^,^^7^ Any potential advantages must be weighed against the more challenging surgery^8^, ^9^, ^10^, ^11^, ^12^ and the unique risks of femoral neck fracture and adverse reactions to metal wear products.^4^^,^^13^

Usage of HRA in Australia has declined since 2005,^14^ following reports of high revision rates in multiple national registries.^8^^,^^15^ These reports included implants withdrawn from the market^4^ and now-contraindicated patients.^15^ For younger men with osteoarthritis, studies report ≥94 % survivorship at 10 years for contemporary HRA prostheses,^5^^,^^14^^,^^16^, ^17^, ^18^, ^19^, ^20^, ^21^ while THA shows a greater revision risk for younger than older patients.^14^ Adverse reactions to metal wear products were prominent causes of revision in the failed designs and remain a leading concern.^14^^,^^22^^,^^23^ These are more frequently observed beyond five years after primary surgery.^14^^,^^24^ Because outcomes are dependent on prosthesis design, metallurgy, size and patient sex,^14^^,^^15^^,^^23^, ^24^, ^25^ evaluation of long-term data for currently available implants is critical.

The aim of this study was to report long-term outcomes for the most frequently used HRA in Australia at present (AHR). Revision rates and causes were compared to the five best-performing conventional THA (5THA). The next most-used and longest-established HRA was used as a class-specific benchmark (BHR). We specifically addressed:

• Does AHR match or exceed the overall survivorship of BHR and 5THA?
• Does AHR show greater survivorship than 5THA for young patients?
• Does AHR show greater survivorship than 5THA for the HRA target cohort of men under 65 years?
• How do the leading causes of revision compare between AHR and benchmark prostheses?

## 2 Patients and methods

### 2.1 Joint replacement registry data

The Australian Orthopaedic Association National Joint Replacement Registry (AOANJRR) has collected data on 98.8 % of hip, knee and shoulder arthroplasty performed in Australia since 2003.^14^ This study included primary total hip procedures undertaken from September 1, 1999 to December 31, 2023, in patients with a primary diagnosis of osteoarthritis (OA). The AOANJRR data collection form presents “male” and “female” options without explanation; we assumed that sex assigned at birth was reported. As all data were obtained in aggregate form, with no identifying information, no Institutional Review Board approval was required. The main outcome considered was the time to first revision.

Procedures were grouped according to five prosthesis combinations. The ADEPT hip resurfacing system (MatOrtho, Leatherhead, United Kingdom) is currently the most commonly used HRA in Australia (AHR).^14^ It has been used in Australia since 2005, and in over half of primary HRA since 2015.^14^^,^^26^^,^^27^ The Birmingham Hip Resurfacing (Smith & Nephew Orthopaedics, Warwick, UK) has the most extensive HRA outcome data in the registry),^14^ so was used as a benchmark HRA (BHR). The five best-performing conventional THA (5THA) were used as an overall benchmark. These were the five cementless prosthesis combinations available and used in 2023 (described as modern prostheses) with the lowest cumulative percent revision (CPR) at 10 years, with head sizes ≥32 mm: Polarstem/EP-Fit Plus (Smith & Nephew, Memphis, TN, USA; 10-year CPR 0.7, 95 % confidence interval (CI), 0.4 to 1.4), Synergy/Reflection (shell) (Smith & Nephew, Memphis, TN, USA; 10-year CPR 2.6, 95 % CI, 2.2 to 3.1), C2/Delta-TT (The LimaCorporate (now Enovis), Udine, Italy; 10-year CPR 3.0, 95 % CI, 2.0 to 4.4), Tri-Fit TS/Trinity (DePuy Synthes, Warsaw, IN, USA; 10-year CPR 3.1, 95 % CI, 2.5 to 3.7), and Secur-Fit Plus/Trident (Shell) (Stryker, MI, USA; 10-year CPR 3.1, 95 % CI, 2.6 to 3.6).

The study population included patients who underwent HRA using either AHR or BHR or conventional THA using one of the 5THA. Bilateral joint replacements were counted separately. As the use of smaller femoral head sizes is no longer recommended,^28^ all BHR and AHR procedures with femoral head diameters below 50 mm were excluded. There were 2439 AHR, 8529 BHR and 17,670 5THA primary procedures for OA during the index time period. The median ages for patients in each group were 54 years for HRA (interquartile range (IQR), 47 to 60 for AHR and 48 to 60 for BHR), and 65 (IQR, 58 to 72) for 5THA (Table A.2). To address differences in the age distributions between HRA and THA, results were additionally stratified by patient age. To limit selection bias, outcomes were further compared for what is generally considered the best indicated sub-group for hip resurfacing arthroplasty: men aged <65 years old. The median follow-up times were AHR 5.2 (IQR 2.7 to 8.2), BHR 15.8 (11.1–19.2), and 5THA 8.4 (5.0–13.9) years.

### 2.2 Data analyses

Kaplan-Meier estimates of survivorship were used to report the time to revision of a HRA, with censoring at the time of death or closure of the dataset at the end of December 2023. The unadjusted CPR, with 95 % confidence intervals (CI), was calculated using unadjusted pointwise Greenwood estimates. Age-adjusted and sex-adjusted hazard ratios (HR) calculated from Cox proportional hazards were checked analytically for each model. If the interaction between the predictor and the log of time was statistically significant in the standard Cox model, then a time varying model was estimated. Time points were selected based on the greatest change in hazard, weighted by a function of events. Time points were iteratively chosen until the assumption of normality was upheld and HRs were calculated for each selected time period. For the present study, if no time period is specified, the HR was calculated over the entire follow-up period. All tests were 2-tailed at 5 % significance levels. Statistical analyses were performed using SAS software version 9.4 (SAS Institute Inc, Cary, NC, USA).

Cumulative incidence functions are used to provide estimates of revision rates in the presence of competing risks (e.g. risk of patient death prior to revision). Plots of cumulative incidence according to revision diagnosis are used to investigate potential failure mechanisms and differences between groups in patterns of revision over time.

## 3 Results and discussion

### 3.1 Revision rates

The CPR at 17 years for the AHR was 5.7 (95 % CI, 3.8 to 8.5), 7.0 (95 % CI, 6.4 to 7.7) for BHR, and 3.9 (95 % CI, 3.5 to 4.3) for the 5THA group (Table A.3). All three groups meet the NICE standard of CPR ≤5 at 10 years.^29^ The study prosthesis had a significantly higher rate of revision compared to 5THA for the entire period (HR 1.43 (95 % CI, 1.06 to 1.92), *P=*0.018). There was no significant difference in the rate of revision between AHR and BHR (Fig. 1).

**Fig. 1:**
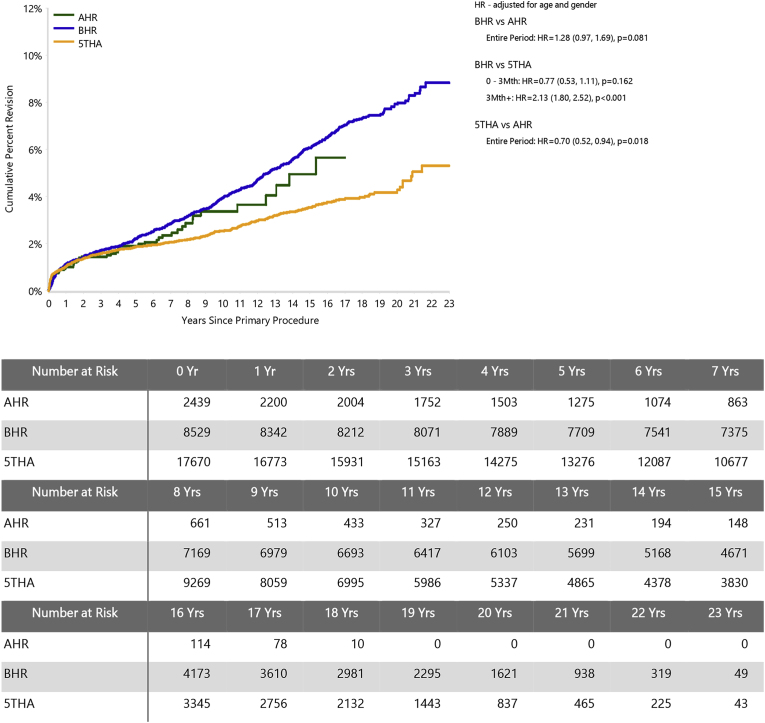
Cumulative percent revision of primary total hip arthroplasty, comparing hip resurfacings A and B (AHR, BHR) and the five best-performing conventional hip arthroplasties (5THA).

Patients with AHR aged <55 years (54.2 % of AHR cohort) were at significantly lower risk of revision than those in the 55 to 64 age group (HR = 0.49 (95 % CI, 0.27 to 0.88), *P=*0.018) and those in the 65–74 age group (HR = 0.31 (95 % CI, 0.15 to 0.64), *P=*0.001) (Figure A.1). The CPR in patients aged under <55 years was 2.1 at 10 years (95 % CI, 1.3 to 3.6), and 2.7 at 15 years (95 % CI, 1.5 to 4.7) (Table A.4). There was no significant difference in revision rates between the 65–74 and 55 to 64 age groups (HR = 1.57 (95 % CI, 0.79 to 3.12), *P=*0.202) (Figure A.1).

In patients aged <55 years, the risks of revision were significantly greater for BHR than AHR (HR = 1.78 (95 % CI, 1.11 to 2.85), *P=*0.017) but similar for 5THA and AHR (HR = 1.13 (95 % CI, 0.67 to 1.91), *P=*0.636) (Fig. 2a). For ages 55–64, there was no significant difference in the risk of revision between BHR and AHR (HR = 0.95 (95 %CI, 0.62 to 1.45); *P=*0.807), while AHR had a significantly higher risk of revision than 5THA after three months (HR = 2.21 (95 %CI, 1.36 to 3.61); *P=*0.001) (Fig. 2b). While AHR numbers for older patients are limited, the comparisons were similar in the 65–74 year group (AHR vs BHR: HR = 01.24 (95 % CI, 0.66 to 2.34), *P=*0.503; AHR vs 5THA: HR = 2.44 (95 % CI, 1.33 to 4.47), *P=*0.004; Fig. 2c).

**Fig. 2:**
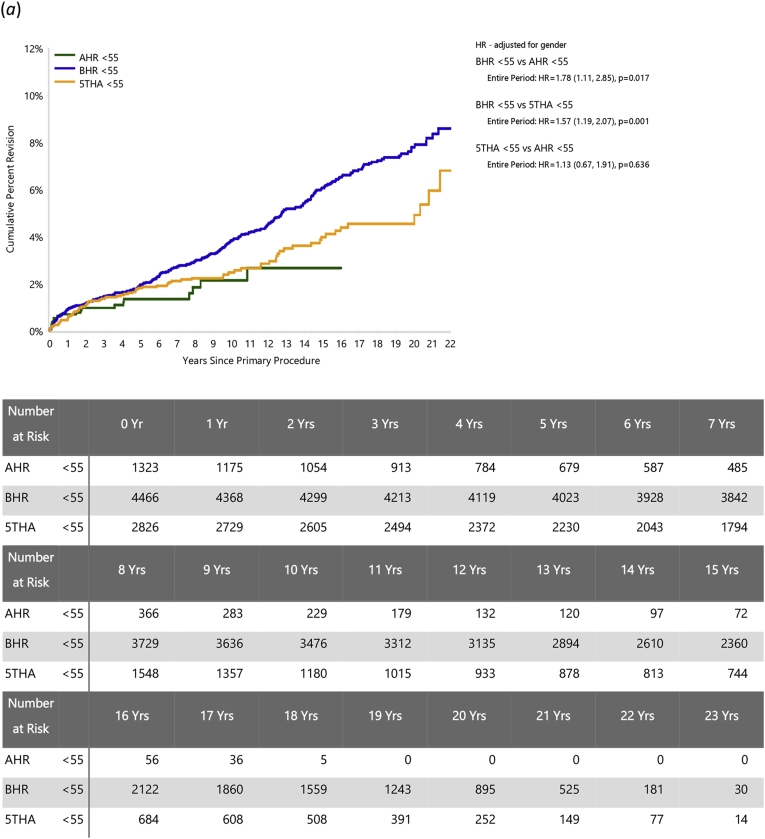

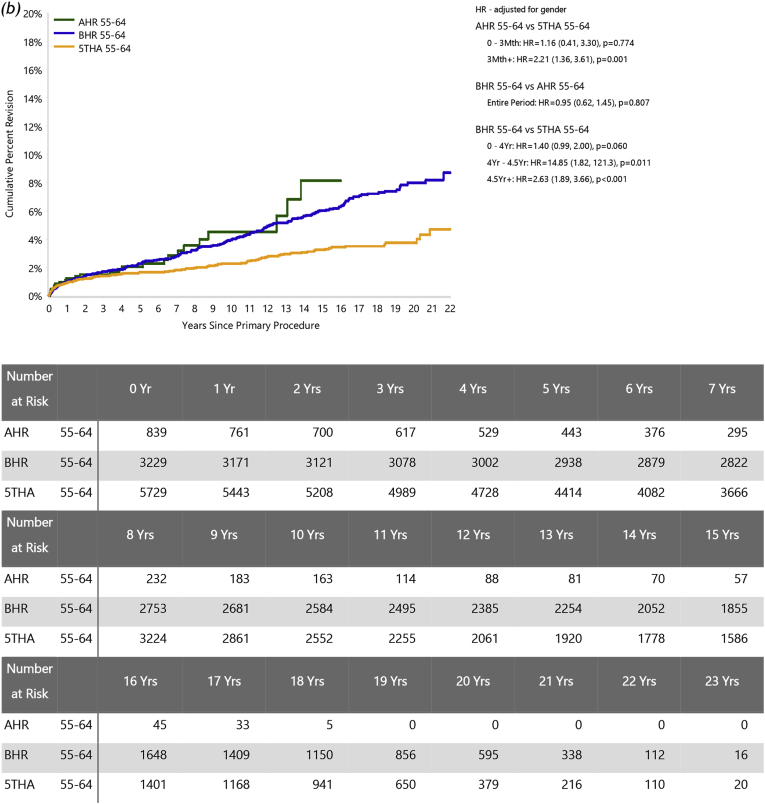

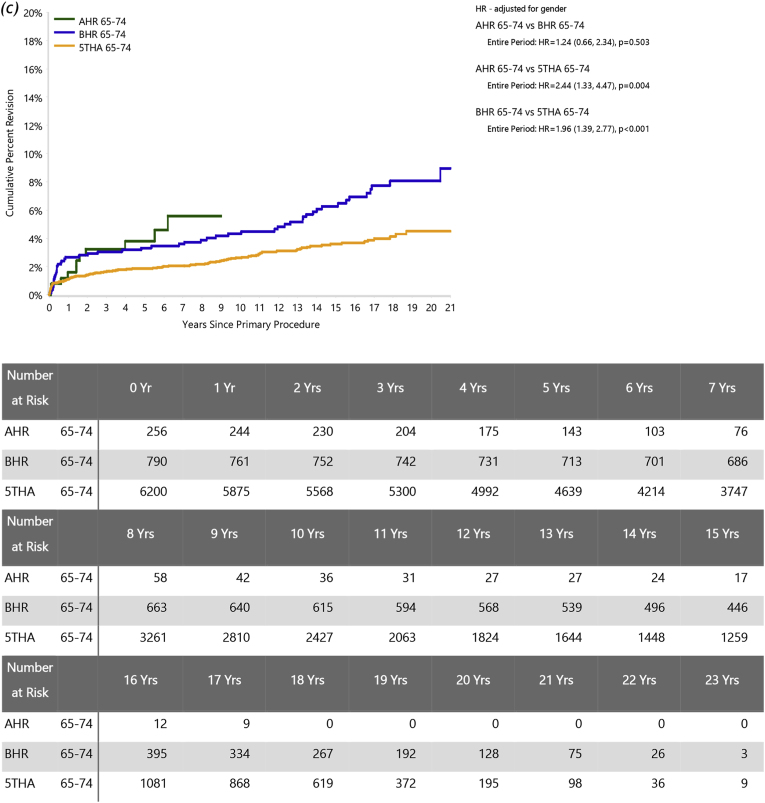
Cumulative percent revision of primary total hip arthroplasty, comparing AHR to BHR and 5THA for each age group. Patients aged (*a*) < 55, (*b*) 55 to 64, (*c*) 65–74 years.

For men aged <65 years, the 17-year CPR was 4.9 (95 % CI, 3.2 to 7.5) for AHR, 6.7 (95 % CI, 6.1 to 7.4) for BHR and 3.8 (95 % CI, 3.1 to 4.6) for the 5THA group (Table A.5). Within this cohort, there was no significant difference in the rate of revision between BHR and AHR (HR = 1.32 (95 % CI, 0.96 to 1.82), *P=*0.085) or between 5THA and AHR (HR = 0.81 (95 % CI, 0.57 to 1.16), *P=*0.250; Fig. 3). While the lower revision risk for AHR patients under 55 years is consistent with its preferential use in younger patients, we found no evidence to support 65 years as an upper age limit for HRA.

**Fig. 3:**
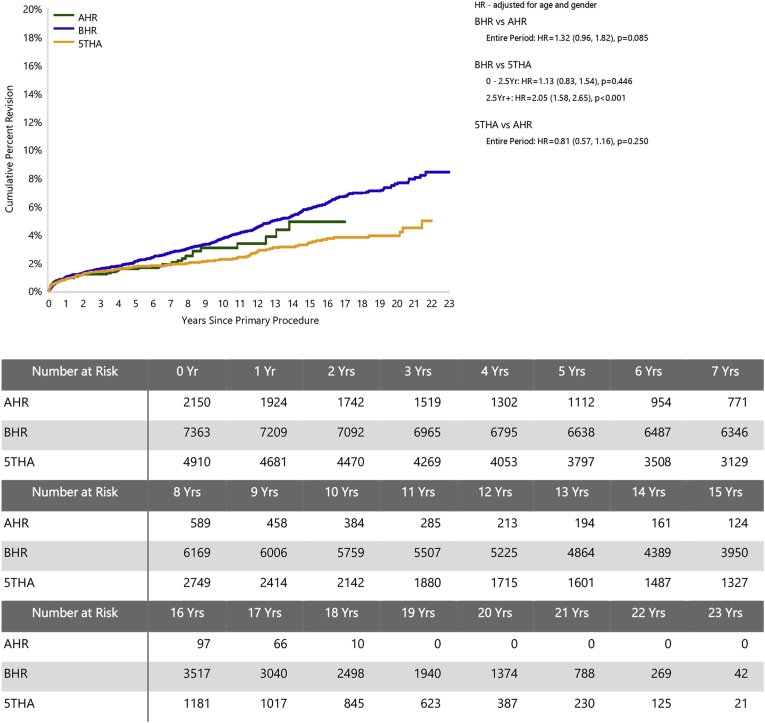
Cumulative percent revision of primary total hip arthroplasty in men aged <65 years, comparing AHR, BHR and 5THA.

### 3.2 Revision diagnoses

The most common reasons for revision (Table A.6) of the AHR were fracture (40.4 % of revisions; 0.9 % of primary resurfacings) and metal-related pathology (21.1 % of revisions; 0.5 % of primaries). The most common reason for AHR revision varied with time (Fig. 4). Revisions due to fracture were most prevalent early, and more likely to occur within the first year than later, while metal related pathology (MRP) was the most common reason for revision after 13 years. The greater likelihood for fracture to occur early is noted in other HRA studies.^5^^,^^11^^,^^17^^,^^30^, ^31^, ^32^, ^33^, ^34^, ^35^ Loosening was the cause of 10.5 % of AHR revisions (0.2 % of primaries). In contrast, loosening was the most common cause of revision for the BHR (26.6 % of revisions; 1.6 % of primary cases), followed by fracture (22.0 % of revisions; 1.3 % of primary cases) and MRP (18.6 % of revisions; 1.1 % of primary cases). Fracture was the most common cause of revision for 5THA (24.3 % of revisions; 0.6 % of primary cases), followed by loosening (22.3 % of revisions; 0.6 % of primary cases) and prosthesis dislocation/instability (20.5 % of revisions; 0.5 % of primary cases). A low number of AHR was revised for infection and revisions due to dislocation/instability were less common than in 5THA; this is consistent with HRA in general.^5^^,^^11^^,^^14^^,^^15^^,^^23^^,^^36^^,^^37^ The infection figures may however include minor 5THA revisions (e.g. head or modular liner exchange) in cases where similar grades of infection would not warrant implant revision in HRA.^38^

**Fig. 4:**
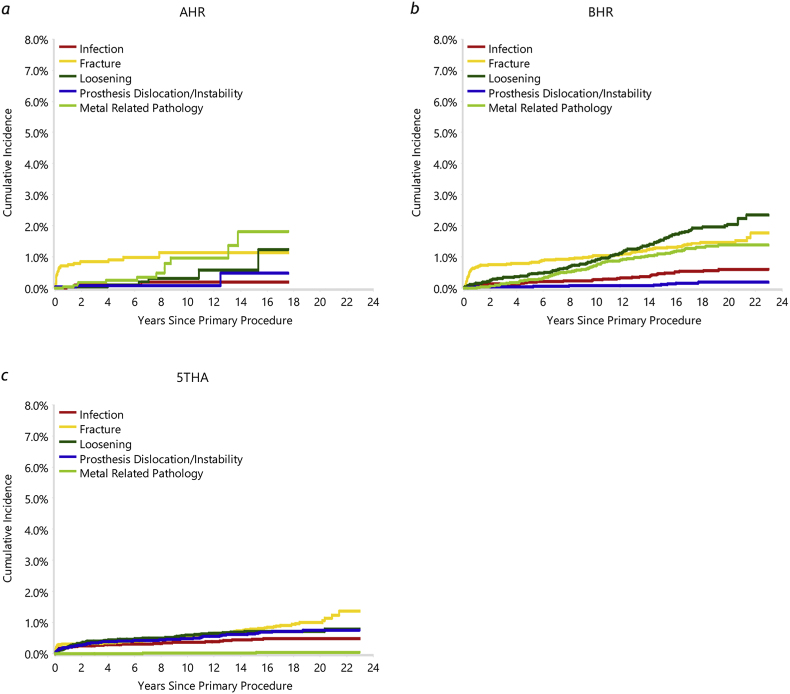
Cumulative incidence of revision diagnoses, for primary (*a*) AHR, (*b*) BHR and (*c*) 5THA.

The rate of revision for fracture was significantly greater for AHR than 5THA (HR = 3.44 (95 % CI, 2.08 to 5.67), *P <* 0.001; Figure A.2a), but there was no difference in the rate of revision compared to BHR (HR = 1.07 (95 % CI, 0.68 to 1.70), *P=*0.766). The prevalence of fracture as a cause for revision was highest in the 65–74 year group for AHR (Figure A.3). An increase with age was also apparent for BHR, in agreement with previous studies^21^^,^^39^ and may reflect a trend in bone quality. Increased periprosthetic fracture incidence among older patients is also apparent in THA patients in general,^14^ although not evident in 5THA (Figure A.3).

With only modern prostheses included in the analysis, 5THA showed only four revisions for MRP (Table A.6). There was no significant difference in the rate of revision for MRP between AHR and BHR (HR = 1.30 (95 % CI, 0.70 to 2.41), *P=*0.401; Figure A.2b), but the cumulative incidence trended higher for AHR (particularly after 13 years; Fig. 4). Metal-related pathology was prominent in the 55–64 year AHR group (Figure A.3), and much less prevalent in younger patients; there are insufficient data to confidently assess the influence of age on this cause of revision. It remains a major concern for metal-on-metal bearings in general, and is associated with excessive wear and therefore more likely beyond five years after primary surgery.^10^^,^^11^^,^^17^^,^^20^^,^^23^^,^^24^^,^^40^ It is a likely underlying cause of osteolysis and pain as revision diagnoses,^17^ although both of these were rare for AHR (Table A.6).

The benchmark hip resurfacing had a significantly higher risk of revision due to loosening (Figure A.2c) compared to AHR (HR = 3.11 (95 % CI, 1.36 to 7.08), *P=*0.006). There was no significant difference in the risk of revision for loosening between 5THA and AHR (HR = 1.83 (95 % CI, 0.78 to 4.28), *P=*0.161). Increased friction due to a wear-related loss of lubrication at the rim of the bearing has been identified as a likely cause of loosening in some studies.^41^^,^^42^ This is consistent with the similar late onset of loosening and MRP in our data (Fig. 4).

### 3.3 Potential limitations

Limitations of the study include its necessarily observational design and the small AHR cohort relative to the comparison groups (see Fig. 1 and Table A.2). Because of the limited numbers, bilateral procedures were considered separately, which risks overweighting outcomes for individual patients. There were only 13 AHR procedures in women, precluding analysis according to patient sex. However, we found no strong justification in the data for excluding these cases. Although HRA revision rates overall are higher for women,^14^ there is a strong bias towards smaller femoral head sizes, which are in turn associated with significantly higher revision rates across HRA generally.^14^^,^^23^ Case numbers for women in the AHR group were too small to test for the independent effects of sex and femoral head size apparent in AOANJRR HRA data.^14^ We note that the AOANJRR does not specify a basis for classifying male and female in patient data, and surgeons and hospitals submit records independently. This may introduce variability due to interpretation at data collection and is not guaranteed to accurately capture sex-specific differences in hip morphology.

To minimise the effect of selection biases, comparisons were restricted to matching primary diagnosis (OA) and comparable contemporary femoral head sizes (≥50 mm for HRA and ≥32 mm for 5THA). Further analyses compared outcomes for the sub-group generally considered suitable for HRA: men aged <65. Because HRA is favoured for younger, more active patients, comparisons with THA may not fully account for differences in the general health and fitness of the cohorts. Although the data have only been collected by the AOANJRR recently, ASA (American Society of Anesthesiologists Physical Status Classification) data suggest that AHR patients are more likely in better health than 5THA patients (ASA ≥3: AHR 12.7 % vs 5THA 22.2 %) while body mass index (BMI) figures were similar (BMI ≥30: AHR 34.1 % vs 5THA 35.8 %; Table A.2). Both ASA and BMI correspond significantly to revision risk in THA.^14^ Further potential selection biases towards THA use include poorer bone quality and hip morphology. Ongoing ASA and BMI data collection will allow further refinement of comparisons.

To limit potential assessment biases in comparing outcomes for a specific prosthesis to all others in a class, outcomes for the AHR were compared to the most established modern HRA prosthesis as a benchmark. Because the comparison prosthesis has been in use for longer, it is possible that its record includes more outcomes influenced by the initial learning curve with HRA. However, we have not investigated the effect of surgeons’ overall HRA experience on prosthesis-specific outcomes. For comparison to conventional THA, the five best-performing (lowest 10-year CPR) prosthesis combinations were selected as an overall best-practice benchmark.

The AOANJRR captures only problems that progress to prosthesis revision or removal. Because clinical evidence does not always correspond to symptoms requiring revision, this likely underestimates the incidence of infection and MRP. Nonetheless, while investigations of metal ions in blood have shown either stabilisation or decreasing levels in patients who have well-functioning HRA prostheses after the first couple of years,^43^, ^44^, ^45^ and a corresponding decrease in the risk of adverse reactions over time,^45^ the present data for both AHR and BHR showed an increasing incidence of revision for MRP (Fig. 3).

Neither the AOANJRR nor the British National Joint Registry capture functional outcomes or radiographic assessments. While it is possible that this masks adverse outcomes that have not yet progressed to revision,^21^ quality of life benefits are likewise unavailable. Reported advantages of HRA over conventional THA include more faithful restoration of biomechanics,^36^^,^^46^ improved function^4^^,^^16^^,^^30^^,^^36^^,^^47^, ^48^, ^49^, ^50^, ^51^ and lower incidence of revision for dislocation.^14^^,^^23^^,^^36^ Although there have been few comparative studies, there is some evidence that HRA patients are more likely to return to high activity levels and may resume high-impact activities.^4^ Analyses to date have found no association between post-operative activity and revision rates^52^ or metal ion levels.^53^^,^^54^

## 4 Conclusions

Australian registry data reveal an estimated survivorship of 94.0 % at 17 years for the HRA most commonly used in Australia since 2015 (AHR), with femoral head sizes ≥50 mm and a primary diagnosis of osteoarthritis. Revision risks for AHR were not significantly different from those of the benchmark HRA (BHR) but higher than the conventional THA benchmark (5THA). Survivorship for patients aged under 55 was 97.3 % at 16 years, and 95.1 % at 17 years for men under 65. For patients under 55 years old, AHR showed a similar risk of revision to 5THA and lower than BHR, whereas AHR patients aged 55 to 64 had a significantly higher revision risk than with 5THA. In the typical HRA cohort of men under 65 years, the revision risk was comparable to both benchmarks. Fracture was the leading cause for AHR revision, its incidence most notable during the first year. Metal-related pathology remains a risk for its metal-on-metal bearing, particularly after eight years and in patients ≥55 years old. Revisions of AHR for infection and dislocation were rare.

## CRediT authorship contribution statement

Cameron Wilson: Conceptualization, Funding acquisition, Investigation, Project administration, Visualization, Writing - original draft, Writing - review & editing.

Michelle Lorimer: Data curation, Formal analysis, Visualization, Writing - review & editing.

Carl Holder: Data curation, Formal analysis, Methodology, Visualization, Writing - review & editing.

James Stoney: Writing - review & editing.

Patrick Weinrauch: Conceptualization, Funding acquisition, Supervision, Writing - review & editing.

## Figures and Tables

**figs1:**
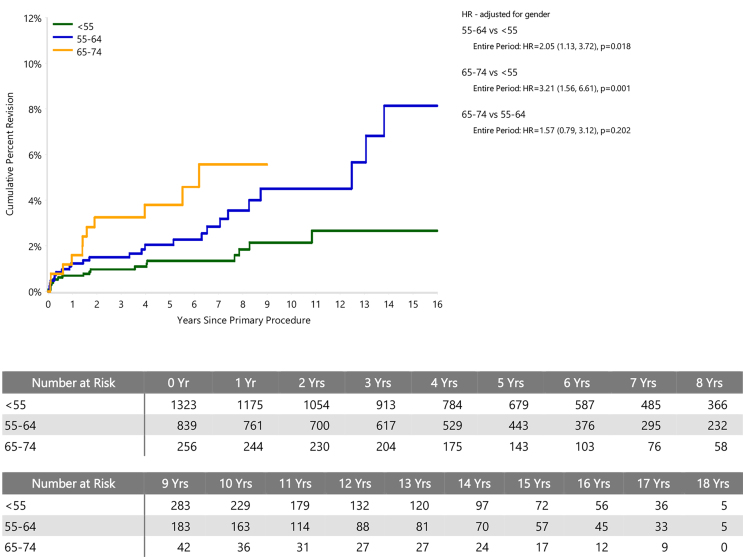


**figs2:**
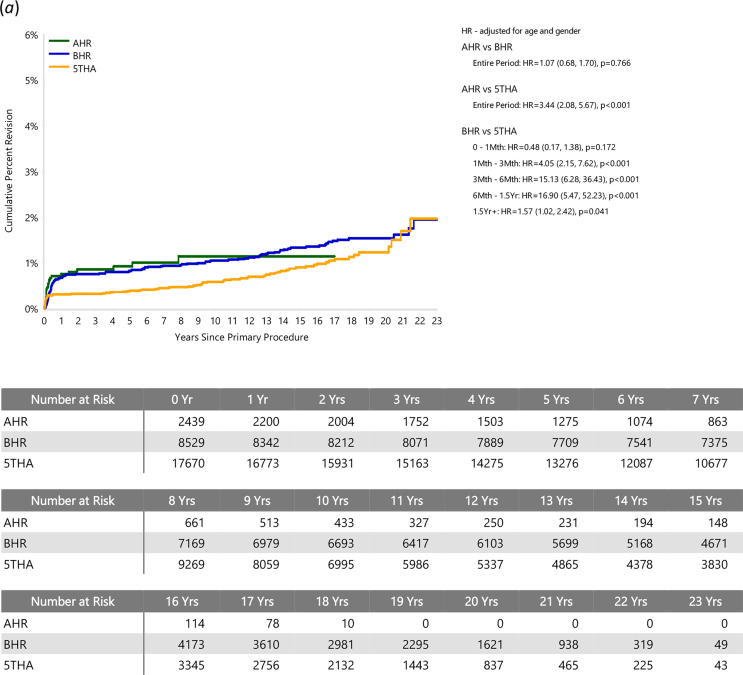


**figs3:**
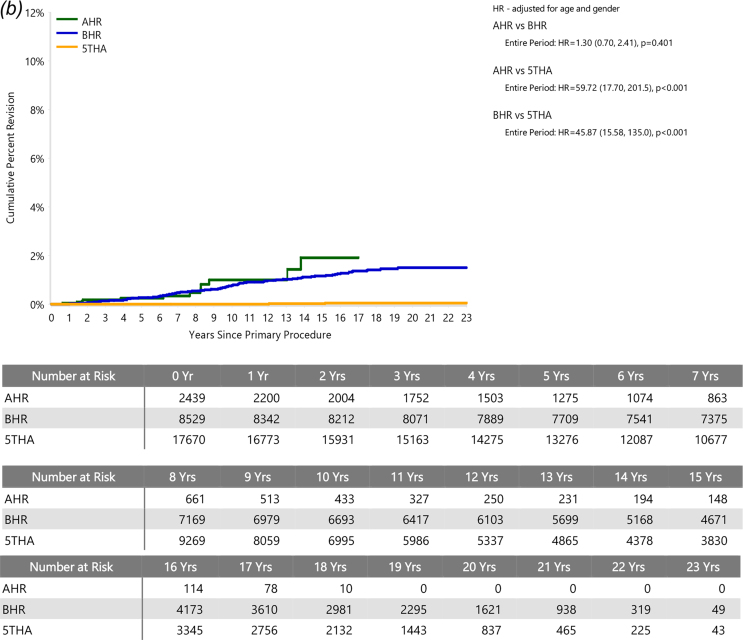


**figs4:**
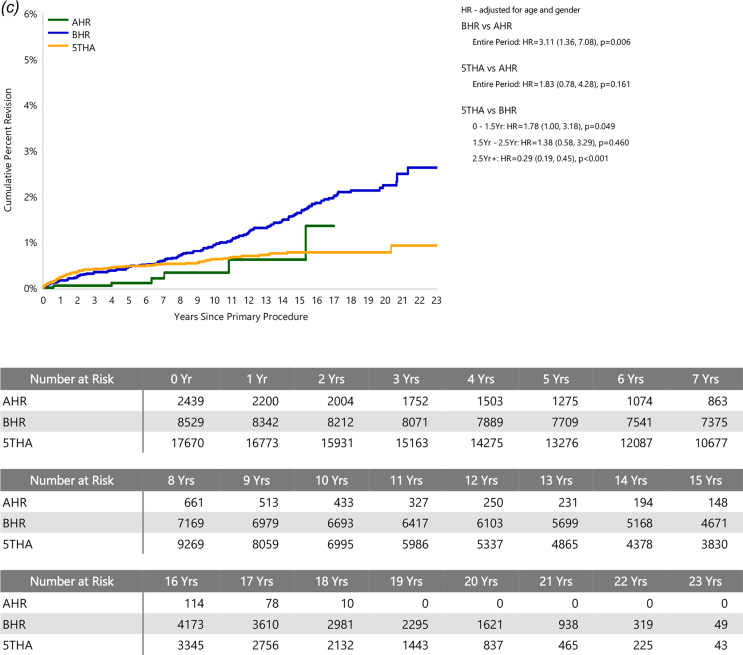


**figs5:**