# Supplementary material for: Seventeen-year outcomes for a contemporary total hip resurfacing prosthesis in Australia: an analysis of registry data with comparison to best performing conventional and most prevalent resurfacing prostheses
Source: J Orthop. 2025 Jul 14;67:299–307. doi: 10.1016/j.jor.2025.07.012 (PMC12302185; doi:10.1016/j.jor.2025.07.012)
Supplement: Multimedia component 1 [file mmc1.docx]

Appendix A. Supplementary Data

## Table Captions

Table A.1: The five best performing conventional primary total hip arthroplasty prosthesis combinations (THA). These had the lowest 10-year cumulative percent revision (CPR) among cementless prostheses with femoral head size ≥32mm, considering only modern prostheses as defined in the 2024 Australian Orthopaedic Association National Joint Replacement Registry Annual Report [14]. 95% confidence intervals for CPR are shown in brackets.

Table A.2: Summary of demographic data for AHR, BHR and 5THA, at the time of primary arthroplasty. Abbreviations: SD - standard deviation, IQR - interquartile range, ASA - American Society of Anesthesiologists, BMI - Body Mass Index (kg/m^2^). ASA^1^ and BMI^2^ figures exclude 15,258 and 17,817 procedures, respectively, for which data were unavailable.

Table A.3: Yearly cumulative percent revision (with 95% confidence intervals) of primary total hip arthroplasty for AHR, BHR and 5THA.

Table A.4: Age-stratified yearly cumulative percent revision (with 95% confidence intervals) of primary total hip arthroplasty for AHR, BHR and 5THA.

Table A.5: Yearly cumulative percent revision (with 95% confidence intervals) of primary total hip arthroplasty for men aged <65 years, comparing AHR, BHR and 5THA.

Table A.6: Revision diagnosis of primary total hip arthroplasty, comparing AHR, BHR and 5THA. This table is restricted to revisions within 18.5 years for all groups, to allow a time-matched comparison of revisions.

## Figure Captions

Figure A.1: Cumulative percent revision of primary total hip resurfacing for AHR, stratified by patient age.

Figure A.2: Cumulative percent revision for (a) fracture, (b) metal-related pathology and (c) loosening, comparing primary AHR, BHR and 5THA.

Figure A.3: Age-stratified cumulative incidence of revision diagnoses, comparing primary AHR, BHR and 5THA. (a–c) ages <55, (d–f) ages 55 to 64, (g–i) ages 65 to 74; (a, d, g) AHR, (b, e, h) BHR, (c, f, i) 5THA.
