# Supplementary material for: Seventeen-year outcomes for a contemporary total hip resurfacing prosthesis in Australia: an analysis of registry data with comparison to best performing conventional and most prevalent resurfacing prostheses
Source: J Orthop. 2025 Jul 14;67:299–307. doi: 10.1016/j.jor.2025.07.012 (PMC12302185; doi:10.1016/j.jor.2025.07.012)
Supplement: Multimedia component 2 [file mmc2.docx]

| ***n*** | **Model** | **10-year CPR** | **Total** |
| --- | --- | --- | --- |
| 1 | Polarstem/EP-Fit Plus | 0.7 (0.4 to 1.4) | 2773 |
| 2 | Synergy/Reflection (Shell) | 2.6 (2.2 to 3.1) | 4614 |
| 3 | C2/Delta-TT | 3.0 (2.0 to 4.4) | 1212 |
| 4 | Tri-Fit TS/Trinity | 3.1 (2.5 to 3.7) | 4832 |
| 5 | Secur-Fit Plus/Trident (Shell) | 3.1 (2.6 to 3.6) | 5273 |
