# Supplementary material for: Seventeen-year outcomes for a contemporary total hip resurfacing prosthesis in Australia: an analysis of registry data with comparison to best performing conventional and most prevalent resurfacing prostheses
Source: J Orthop. 2025 Jul 14;67:299–307. doi: 10.1016/j.jor.2025.07.012 (PMC12302185; doi:10.1016/j.jor.2025.07.012)
Supplement: Multimedia component 3 [file mmc3.docx]

| **Variable** |  | **AHR** | **BHR** | **5THA** | **TOTAL** |
| --- | --- | --- | --- | --- | --- |
| **Follow Up Years** |  |  |  |  |  |
|  | Mean ± SD | 6 ± 4.4 | 14.6 ± 5.8 | 9.4 ± 5.9 | 10.7 ± 6.4 |
|  | Median (IQR) | 5.2 (2.7 to 8.2) | 15.8 (11.1 to 19.2) | 8.4 (5 to 13.9) | 9.8 (5.5 to 16.4) |
|  | Minimum | 0 | 0 | 0 | 0 |
|  | Maximum | 18.5 | 23.7 | 23.7 | 23.7 |
| **Age** |  |  |  |  |  |
|  | Mean ± SD | 53.3 ± 9.4 | 53.5 ± 8.8 | 64.5 ± 10.1 | 60.3 ± 11.1 |
|  | Median (IQR) | 54 (47 to 60) | 54 (48 to 60) | 65 (58 to 72) | 60 (53 to 68) |
| **Sex** |  |  |  |  |  |
|  | Male | 2,426 (99.5%) | 8,172 (95.8%) | 9,750 (55.2%) | 20,348 (71.1%) |
|  | Female | 13 (0.5%) | 357 (4.2%) | 7,920 (44.8%) | 8,290 (28.9%) |
| **ASA Score^1^** |  |  |  |  |  |
|  | 1 | 632 (30.7%) | 610 (42%) | 1,383 (14%) | 2,625 (19.6%) |
|  | 2 | 1,164 (56.6%) | 729 (50.2%) | 6,300 (63.8%) | 8,193 (61.2%) |
|  | 3 | 248 (12.1%) | 112 (7.7%) | 2,121 (21.5%) | 2,481 (18.5%) |
|  | 4 | 13 (0.6%) |  | 68 (0.7%) | 81 (0.6%) |
| **BMI^2^** |  |  |  |  |  |
|  | Underweight (<18.50) | 1 (0.1%) | 1 (0.1%) | 40 (0.5%) | 42 (0.4%) |
|  | Normal (18.50 to 24.99) | 305 (16.9%) | 163 (16.1%) | 1,803 (22.5%) | 2,271 (21%) |
|  | Pre Obese (25.00 to 29.99) | 882 (48.9%) | 490 (48.3%) | 3,300 (41.2%) | 4,672 (43.2%) |
|  | Obese Class 1 (30.00 to 34.99) | 457 (25.4%) | 253 (25%) | 1,919 (24%) | 2,629 (24.3%) |
|  | Obese Class 2 (35.00 to 39.99) | 122 (6.8%) | 78 (7.7%) | 680 (8.5%) | 880 (8.1%) |
|  | Obese Class 3 (≥40.00) | 35 (1.9%) | 29 (2.9%) | 263 (3.3%) | 327 (3%) |
| **TOTAL** |  | 2,439 | 8,529 | 17,670 | 28,638 |
