# Supplementary material for: Seventeen-year outcomes for a contemporary total hip resurfacing prosthesis in Australia: an analysis of registry data with comparison to best performing conventional and most prevalent resurfacing prostheses
Source: J Orthop. 2025 Jul 14;67:299–307. doi: 10.1016/j.jor.2025.07.012 (PMC12302185; doi:10.1016/j.jor.2025.07.012)
Supplement: Multimedia component 4 [file mmc4.docx]

| **CPR** | **1 Yr** | **2 Yrs** | **3 Yrs** | **4 Yrs** | **5 Yrs** | **6 Yrs** |
| --- | --- | --- | --- | --- | --- | --- |
| AHR | 1.0 (0.7 to 1.5) | 1.4 (1.0 to 2.0) | 1.4 (1.0 to 2.0) | 1.8 (1.3 to 2.4) | 1.9 (1.4 to 2.6) | 2.1 (1.5 to 2.8) |
| BHR | 1.1 (0.9 to 1.4) | 1.4 (1.2 to 1.7) | 1.7 (1.5 to 2.0) | 1.9 (1.6 to 2.2) | 2.2 (1.9 to 2.6) | 2.5 (2.2 to 2.9) |
| 5THA | 1.0 (0.9 to 1.2) | 1.4 (1.2 to 1.6) | 1.6 (1.4 to 1.8) | 1.7 (1.6 to 2.0) | 1.9 (1.7 to 2.1) | 2.0 (1.8 to 2.2) |
| **CPR** | **7 Yrs** | **8 Yrs** | **9 Yrs** | **10 Yrs** | **11 Yrs** | **12 Yrs** |
| AHR | 2.4 (1.7 to 3.2) | 2.9 (2.1 to 3.9) | 3.4 (2.5 to 4.6) | 3.4 (2.5 to 4.6) | 3.7 (2.6 to 5.0) | 3.7 (2.6 to 5.0) |
| BHR | 2.9 (2.5 to 3.2) | 3.2 (2.8 to 3.6) | 3.5 (3.1 to 3.9) | 4.0 (3.5 to 4.4) | 4.3 (3.9 to 4.8) | 4.7 (4.3 to 5.2) |
| 5THA | 2.1 (1.9 to 2.3) | 2.2 (2.0 to 2.4) | 2.3 (2.1 to 2.6) | 2.5 (2.3 to 2.8) | 2.8 (2.5 to 3.1) | 3.0 (2.7 to 3.3) |
| **CPR** | **13 Yrs** | **14 Yrs** | **15 Yrs** | **16 Yrs** | **17 Yrs** | **18 Yrs** |
| AHR | 4.1 (2.9 to 5.7) | 4.9 (3.4 to 7.2) | 4.9 (3.4 to 7.2) | 5.7 (3.8 to 8.5) | 5.7 (3.8 to 8.5) |  |
| BHR | 5.2 (4.7 to 5.7) | 5.6 (5.1 to 6.1) | 6.1 (5.5 to 6.7) | 6.5 (5.9 to 7.1) | 7.0 (6.4 to 7.7) | 7.3 (6.7 to 8.0) |
| 5THA | 3.2 (2.9 to 3.6) | 3.4 (3.0 to 3.7) | 3.6 (3.2 to 3.9) | 3.8 (3.4 to 4.2) | 3.9 (3.5 to 4.3) | 4.0 (3.6 to 4.4) |
| **CPR** | **19 Yrs** | **20 Yrs** | **21 Yrs** | **22 Yrs** | **23 Yrs** |  |
| AHR |  |  |  |  |  |  |
| BHR | 7.5 (6.8 to 8.2) | 7.9 (7.2 to 8.7) | 8.3 (7.5 to 9.1) | 8.8 (7.9 to 9.8) | 8.8 (7.9 to 9.8) |  |
| 5THA | 4.2 (3.7 to 4.7) | 4.2 (3.7 to 4.7) | 5.1 (4.3 to 6.0) | 5.3 (4.4 to 6.4) | 5.3 (4.4 to 6.4) |  |
