# Supplementary material for: Seventeen-year outcomes for a contemporary total hip resurfacing prosthesis in Australia: an analysis of registry data with comparison to best performing conventional and most prevalent resurfacing prostheses
Source: J Orthop. 2025 Jul 14;67:299–307. doi: 10.1016/j.jor.2025.07.012 (PMC12302185; doi:10.1016/j.jor.2025.07.012)
Supplement: Multimedia component 5 [file mmc5.docx]

| **Model** | **Age** | **1 Yr** | **2 Yrs** | | **3 Yrs** | | **4 Yrs** | | **5 Yrs** | | **6 Yrs** | | **7 Yrs** | | **8 Yrs** |
| --- | --- | --- | --- | --- | --- | --- | --- | --- | --- | --- | --- | --- | --- | --- | --- |
| AHR | <55 | 0.7 (0.4 to 1.3) | 1.0 (0.6 to 1.7) | | 1.0 (0.6 to 1.7) | | 1.1 (0.6 to 1.9) | | 1.3 (0.8 to 2.2) | | 1.3 (0.8 to 2.2) | | 1.3 (0.8 to 2.2) | | 1.9 (1.1 to 3.1) |
|  | 55 to 64 | 1.2 (0.7 to 2.3) | 1.5 (0.9 to 2.6) | | 1.5 (0.9 to 2.6) | | 2.0 (1.2 to 3.4) | | 2.0 (1.2 to 3.4) | | 2.3 (1.4 to 3.7) | | 2.9 (1.8 to 4.6) | | 3.6 (2.2 to 5.7) |
|  | 65 to 74 | 1.6 (0.6 to 4.2) | 3.2 (1.6 to 6.4) | | 3.2 (1.6 to 6.4) | | 3.8 (2.0 to 7.2) | | 3.8 (2.0 to 7.2) | | 4.6 (2.4 to 8.5) | | 5.6 (3.0 to 10.2) | | 5.6 (3.0 to 10.2) |
|  | ≥75 | 4.8 (0.7 to 29.3) | 4.8 (0.7 to 29.3) | | 4.8 (0.7 to 29.3) | | 4.8 (0.7 to 29.3) | | 4.8 (0.7 to 29.3) | | 4.8 (0.7 to 29.3) | | 4.8 (0.7 to 29.3) | | 4.8 (0.7 to 29.3) |
| BHR | <55 | 0.9 (0.7 to 1.3) | 1.2 (0.9 to 1.5) | | 1.5 (1.1 to 1.9) | | 1.6 (1.3 to 2.1) | | 2.0 (1.6 to 2.4) | | 2.3 (1.9 to 2.8) | | 2.7 (2.3 to 3.2) | | 3.0 (2.5 to 3.5) |
|  | 55 to 64 | 1.0 (0.7 to 1.4) | 1.4 (1.0 to 1.9) | | 1.7 (1.3 to 2.2) | | 1.9 (1.5 to 2.4) | | 2.3 (1.8 to 2.9) | | 2.5 (2.0 to 3.2) | | 2.8 (2.3 to 3.5) | | 3.2 (2.7 to 3.9) |
|  | 65 to 74 | 2.7 (1.7 to 4.1) | 2.8 (1.8 to 4.2) | | 3.1 (2.1 to 4.5) | | 3.2 (2.2 to 4.7) | | 3.3 (2.3 to 4.8) | | 3.5 (2.4 to 5.0) | | 3.6 (2.5 to 5.2) | | 3.9 (2.7 to 5.5) |
|  | ≥75 | 4.5 (1.2 to 17.0) | 4.5 (1.2 to 17.0) | | 4.5 (1.2 to 17.0) | | 4.5 (1.2 to 17.0) | | 4.5 (1.2 to 17.0) | | 4.5 (1.2 to 17.0) | | 8.2 (2.6 to 24.1) | | 8.2 (2.6 to 24.1) |
| 5THA | <55 | 0.5 (0.3 to 0.9) | 1.1 (0.8 to 1.6) | | 1.4 (1.0 to 1.9) | | 1.5 (1.1 to 2.1) | | 1.8 (1.4 to 2.4) | | 1.9 (1.5 to 2.5) | | 2.1 (1.6 to 2.8) | | 2.2 (1.7 to 2.9) |
|  | 55 to 64 | 0.9 (0.7 to 1.2) | 1.2 (1.0 to 1.5) | | 1.4 (1.1 to 1.8) | | 1.6 (1.3 to 1.9) | | 1.7 (1.3 to 2.0) | | 1.7 (1.4 to 2.1) | | 1.8 (1.5 to 2.2) | | 2.0 (1.6 to 2.4) |
|  | 65 to 74 | 1.1 (0.9 to 1.4) | 1.4 (1.1 to 1.7) | | 1.7 (1.4 to 2.0) | | 1.8 (1.5 to 2.2) | | 1.9 (1.5 to 2.2) | | 2.0 (1.7 to 2.4) | | 2.0 (1.7 to 2.4) | | 2.2 (1.8 to 2.6) |
|  | ≥75 | 1.6 (1.2 to 2.1) | 1.9 (1.4 to 2.4) | | 2.0 (1.5 to 2.6) | | 2.2 (1.7 to 2.8) | | 2.3 (1.8 to 3.0) | | 2.5 (2.0 to 3.1) | | 2.5 (2.0 to 3.2) | | 2.5 (2.0 to 3.2) |
| **Model** | **Age** | **9 Yrs** | **10 Yrs** | | **11 Yrs** | | **12 Yrs** | | **13 Yrs** | | **14 Yrs** | | **15 Yrs** | | **16 Yrs** |
| AHR | <55 | 2.1 (1.3 to 3.6) | 2.1 (1.3 to 3.6) | | 2.7 (1.5 to 4.7) | | 2.7 (1.5 to 4.7) | | 2.7 (1.5 to 4.7) | | 2.7 (1.5 to 4.7) | | 2.7 (1.5 to 4.7) | | 2.7 (1.5 to 4.7) |
|  | 55 to 64 | 4.5 (2.8 to 7.2) | 4.5 (2.8 to 7.2) | | 4.5 (2.8 to 7.2) | | 4.5 (2.8 to 7.2) | | 5.7 (3.3 to 9.7) | | 8.1 (4.6 to 14.1) | | 8.1 (4.6 to 14.1) | | 8.1 (4.6 to 14.1) |
|  | 65 to 74 | 5.6 (3.0 to 10.2) |  | |  | |  | |  | |  | |  | |  |
|  | ≥75 | 4.8 (0.7 to 29.3) | 4.8 (0.7 to 29.3) | |  | |  | |  | |  | |  | |  |
| BHR | <55 | 3.3 (2.8 to 3.9) | 3.8 (3.3 to 4.5) | | 4.2 (3.6 to 4.9) | | 4.5 (3.9 to 5.2) | | 5.2 (4.5 to 5.9) | | 5.4 (4.7 to 6.2) | | 6.1 (5.3 to 6.9) | | 6.5 (5.8 to 7.4) |
|  | 55 to 64 | 3.6 (3.0 to 4.3) | 4.0 (3.3 to 4.7) | | 4.4 (3.7 to 5.2) | | 4.9 (4.2 to 5.8) | | 5.2 (4.4 to 6.0) | | 5.6 (4.9 to 6.6) | | 6.0 (5.2 to 7.0) | | 6.3 (5.4 to 7.3) |
|  | 65 to 74 | 4.2 (3.0 to 5.9) | 4.3 (3.1 to 6.0) | | 4.5 (3.2 to 6.2) | | 4.8 (3.5 to 6.6) | | 5.2 (3.8 to 7.0) | | 5.9 (4.4 to 7.9) | | 6.3 (4.7 to 8.4) | | 6.9 (5.2 to 9.2) |
|  | ≥75 | 8.2 (2.6 to 24.1) | 8.2 (2.6 to 24.1) | | 8.2 (2.6 to 24.1) | | 8.2 (2.6 to 24.1) | | 8.2 (2.6 to 24.1) | | 8.2 (2.6 to 24.1) | | 8.2 (2.6 to 24.1) | | 18.4 (5.7 to 50.4) |
| 5THA | <55 | 2.2 (1.7 to 2.9) | 2.5 (1.9 to 3.2) | | 2.7 (2.0 to 3.4) | | 2.9 (2.2 to 3.7) | | 3.5 (2.7 to 4.5) | | 3.6 (2.8 to 4.7) | | 4.0 (3.1 to 5.1) | | 4.4 (3.4 to 5.7) |
|  | 55 to 64 | 2.1 (1.8 to 2.6) | 2.3 (1.9 to 2.8) | | 2.5 (2.0 to 3.0) | | 2.8 (2.3 to 3.3) | | 3.0 (2.5 to 3.6) | | 3.1 (2.6 to 3.7) | | 3.2 (2.7 to 3.9) | | 3.4 (2.9 to 4.1) |
|  | 65 to 74 | 2.4 (2.0 to 2.8) | 2.6 (2.2 to 3.1) | | 2.9 (2.4 to 3.4) | | 3.1 (2.6 to 3.7) | | 3.2 (2.7 to 3.8) | | 3.5 (2.9 to 4.1) | | 3.6 (3.0 to 4.3) | | 3.7 (3.1 to 4.4) |
|  | ≥75 | 2.6 (2.1 to 3.3) | 2.9 (2.3 to 3.7) | | 3.2 (2.5 to 4.1) | | 3.2 (2.5 to 4.1) | | 3.2 (2.5 to 4.1) | | 3.2 (2.5 to 4.1) | | 3.2 (2.5 to 4.1) | | 3.7 (2.6 to 5.2) |
| **Model** | **Age** | **17 Yrs** | | **18 Yrs** | | **19 Yrs** | | **20 Yrs** | | **21 Yrs** | | **22 Yrs** | | **23 Yrs** | |
| AHR | <55 |  | |  | |  | |  | |  | |  | |  | |
|  | 55 to 64 |  | |  | |  | |  | |  | |  | |  | |
|  | 65 to 74 |  | |  | |  | |  | |  | |  | |  | |
|  | ≥75 |  | |  | |  | |  | |  | |  | |  | |
| BHR | <55 | 6.9 (6.0 to 7.8) | | 7.2 (6.4 to 8.2) | | 7.4 (6.5 to 8.3) | | 7.8 (6.9 to 8.9) | | 8.2 (7.2 to 9.3) | | 8.6 (7.5 to 9.9) | |  | |
|  | 55 to 64 | 7.0 (6.1 to 8.1) | | 7.2 (6.3 to 8.3) | | 7.4 (6.4 to 8.5) | | 8.0 (6.9 to 9.2) | | 8.2 (7.1 to 9.5) | | 8.7 (7.3 to 10.4) | |  | |
|  | 65 to 74 | 7.7 (5.9 to 10.2) | | 8.1 (6.1 to 10.6) | | 8.1 (6.1 to 10.6) | | 8.1 (6.1 to 10.6) | | 9.0 (6.5 to 12.2) | |  | |  | |
|  | ≥75 | 18.4 (5.7 to 50.4) | |  | |  | |  | |  | |  | |  | |
| 5THA | <55 | 4.5 (3.5 to 5.8) | | 4.5 (3.5 to 5.8) | | 4.5 (3.5 to 5.8) | | 4.5 (3.5 to 5.8) | | 6.0 (4.3 to 8.3) | | 6.8 (4.7 to 9.9) | |  | |
|  | 55 to 64 | 3.5 (2.9 to 4.2) | | 3.5 (2.9 to 4.2) | | 3.7 (3.1 to 4.5) | | 3.7 (3.1 to 4.5) | | 4.7 (3.6 to 6.2) | | 4.7 (3.6 to 6.2) | |  | |
|  | 65 to 74 | 3.9 (3.2 to 4.7) | | 4.1 (3.4 to 5.0) | | 4.5 (3.7 to 5.6) | | 4.5 (3.7 to 5.6) | | 4.5 (3.7 to 5.6) | |  | |  | |
|  | ≥75 | 3.7 (2.6 to 5.2) | | 3.7 (2.6 to 5.2) | |  | |  | |  | |  | |  | |
