# Supplementary material for: Seventeen-year outcomes for a contemporary total hip resurfacing prosthesis in Australia: an analysis of registry data with comparison to best performing conventional and most prevalent resurfacing prostheses
Source: J Orthop. 2025 Jul 14;67:299–307. doi: 10.1016/j.jor.2025.07.012 (PMC12302185; doi:10.1016/j.jor.2025.07.012)
Supplement: Multimedia component 6 [file mmc6.docx]

| **CPR** | | **1 Yr** | | **2 Yrs** | | **3 Yrs** | | **4 Yrs** | | **5 Yrs** | | **6 Yrs** | |
| --- | --- | --- | --- | --- | --- | --- | --- | --- | --- | --- | --- | --- | --- |
| AHR | | 0.9 (0.6 to 1.4) | | 1.2 (0.8 to 1.8) | | 1.2 (0.8 to 1.8) | | 1.5 (1.0 to 2.1) | | 1.6 (1.1 to 2.2) | | 1.6 (1.1 to 2.4) | |
| BHR | | 1.0 (0.8 to 1.2) | | 1.3 (1.0 to 1.6) | | 1.6 (1.3 to 1.9) | | 1.8 (1.5 to 2.1) | | 2.1 (1.8 to 2.4) | | 2.4 (2.0 to 2.8) | |
| 5THA | | 0.8 (0.6 to 1.1) | | 1.2 (0.9 to 1.5) | | 1.4 (1.1 to 1.7) | | 1.6 (1.2 to 2.0) | | 1.7 (1.4 to 2.1) | | 1.8 (1.4 to 2.2) | |
| **CPR** | | **7 Yrs** | | **8 Yrs** | | **9 Yrs** | | **10 Yrs** | | **11 Yrs** | | **12 Yrs** | |
| AHR | | 1.9 (1.3 to 2.7) | | 2.5 (1.7 to 3.5) | | 3.0 (2.1 to 4.3) | | 3.0 (2.1 to 4.3) | | 3.3 (2.3 to 4.8) | | 3.3 (2.3 to 4.8) | |
| BHR | | 2.7 (2.4 to 3.1) | | 3.0 (2.6 to 3.4) | | 3.3 (2.9 to 3.7) | | 3.7 (3.3 to 4.2) | | 4.1 (3.7 to 4.6) | | 4.5 (4.0 to 5.1) | |
| 5THA | | 1.9 (1.5 to 2.3) | | 2.0 (1.6 to 2.4) | | 2.1 (1.7 to 2.6) | | 2.2 (1.8 to 2.7) | | 2.4 (1.9 to 2.9) | | 2.8 (2.3 to 3.4) | |
| **CPR** | | **13 Yrs** | | **14 Yrs** | | **15 Yrs** | | **16 Yrs** | | **17 Yrs** | | **18 Yrs** | |
| AHR | | 3.8 (2.5 to 5.7) | | 4.9 (3.2 to 7.5) | | 4.9 (3.2 to 7.5) | | 4.9 (3.2 to 7.5) | | 4.9 (3.2 to 7.5) | |  | |
| BHR | | 5.0 (4.5 to 5.6) | | 5.3 (4.8 to 5.9) | | 5.8 (5.3 to 6.5) | | 6.2 (5.6 to 6.9) | | 6.7 (6.1 to 7.4) | | 7.0 (6.3 to 7.7) | |
| 5THA | | 3.1 (2.5 to 3.7) | | 3.1 (2.6 to 3.8) | | 3.4 (2.8 to 4.2) | | 3.7 (3.0 to 4.5) | | 3.8 (3.1 to 4.6) | | 3.8 (3.1 to 4.6) | |
| **CPR** | | **19 Yrs** | | **20 Yrs** | | **21 Yrs** | | **22 Yrs** | | **23 Yrs** | |  | |
| AHR | |  | |  | |  | |  | |  | |  | |
| BHR | | 7.1 (6.4 to 7.8) | | 7.6 (6.9 to 8.4) | | 7.9 (7.1 to 8.8) | | 8.4 (7.5 to 9.5) | | 8.4 (7.5 to 9.5) | |  | |
| 5THA | | 3.9 (3.2 to 4.8) | | 3.9 (3.2 to 4.8) | | 4.5 (3.5 to 5.7) | | 5.0 (3.7 to 6.7) | |  | |  | |
