# Supplementary material for: Seventeen-year outcomes for a contemporary total hip resurfacing prosthesis in Australia: an analysis of registry data with comparison to best performing conventional and most prevalent resurfacing prostheses
Source: J Orthop. 2025 Jul 14;67:299–307. doi: 10.1016/j.jor.2025.07.012 (PMC12302185; doi:10.1016/j.jor.2025.07.012)
Supplement: Multimedia component 7 [file mmc7.docx]

|  | **AHR** | | | **BHR** | | | **5THA** | | |
| --- | --- | --- | --- | --- | --- | --- | --- | --- | --- |
| **Revision Diagnosis** | **Number** | **% Primaries Revised** | **% Revisions** | **Number** | **% Primaries Revised** | **% Revisions** | **Number** | **% Primaries Revised** | **% Revisions** |
| Loosening | 6 | 0.2 | 10.5 | 133 | 1.6 | 26.6 | 100 | 0.6 | 22.3 |
| Fracture | 23 | 0.9 | 40.4 | 110 | 1.3 | 22.0 | 109 | 0.6 | 24.3 |
| Metal Related Pathology | 12 | 0.5 | 21.1 | 93 | 1.1 | 18.6 | 4 | 0.0 | 0.9 |
| Prosthesis Dislocation/ Instability | 3 | 0.1 | 5.3 | 12 | 0.1 | 2.4 | 92 | 0.5 | 20.5 |
| Infection | 3 | 0.1 | 5.3 | 39 | 0.5 | 7.8 | 66 | 0.4 | 14.7 |
| Lysis | 1 | 0.0 | 1.8 | 42 | 0.5 | 8.4 | 9 | 0.1 | 2.0 |
| Pain | 2 | 0.1 | 3.5 | 28 | 0.3 | 5.6 | 18 | 0.1 | 4.0 |
| Osteonecrosis | 2 | 0.1 | 3.5 | 14 | 0.2 | 2.8 |  |  |  |
| Malposition | 2 | 0.1 | 3.5 | 12 | 0.1 | 2.4 | 9 | 0.1 | 2.0 |
| Leg Length Discrepancy | 1 | 0.0 | 1.8 | 1 | 0.0 | 0.2 | 7 | 0.0 | 1.6 |
| Implant Breakage, Acetabular |  |  |  | 1 | 0.0 | 0.2 | 6 | 0.0 | 1.3 |
| Implant Breakage, Acetabular Insert |  |  |  |  |  |  | 6 | 0.0 | 1.3 |
| Progression Of Disease |  |  |  | 4 | 0.0 | 0.8 |  |  |  |
| Wear, Acetabular Insert |  |  |  |  |  |  | 4 | 0.0 | 0.9 |
| Implant Breakage, Head |  |  |  |  |  |  | 2 | 0.0 | 0.4 |
| Incorrect Sizing |  |  |  | 1 | 0.0 | 0.2 | 2 | 0.0 | 0.4 |
| Tumour |  |  |  | 2 | 0.0 | 0.4 | 2 | 0.0 | 0.4 |
| Wear, Head |  |  |  |  |  |  | 2 | 0.0 | 0.4 |
| Heterotopic Bone |  |  |  | 1 | 0.0 | 0.2 |  |  |  |
| Implant Breakage, Stem |  |  |  |  |  |  | 1 | 0.0 | 0.2 |
| Synovitis |  |  |  | 1 | 0.0 | 0.2 |  |  |  |
| Wear, Acetabulum |  |  |  | 1 | 0.0 | 0.2 |  |  |  |
| Other | 2 | 0.1 | 3.5 | 5 | 0.1 | 1.0 | 10 | 0.1 | 2.2 |
| **N Revision** | **57** | **2.3** | **100.0** | **500** | **5.9** | **100.0** | **449** | **2.5** | **100.0** |
| **N Primary** | **2439** |  |  | **8529** |  |  | **17670** |  |  |
